# Sudden Onset of Broad Complex Tachycardia in a Fit Young Man: A Case Report

**DOI:** 10.7759/cureus.50425

**Published:** 2023-12-13

**Authors:** Ayobami B Omodara, Olusegun Areo, Joanita Kintu, Mia Thornton

**Affiliations:** 1 General Internal Medicine, Manchester University NHS Foundation Trust, Manchester, GBR; 2 Cardiology, Tameside and Glossop Integrated Care NHS Foundation Trust, Manchester, GBR; 3 Haematology, The Christie Hospital, Manchester, GBR

**Keywords:** adult tachycardia, accessory pathway, electrophysiological study, broad complex tachycardia, electro cardioversion, wolf-parkinson-white

## Abstract

Wolff-Parkinson-White (WPW) syndrome is a clinical pre-excitation syndrome often strongly associated with tachyarrhythmias that are predominantly atrioventricular re-entrant tachycardia (AVRT). It is generally considered to be a relatively benign arrhythmogenic condition associated with a slightly higher risk of sudden cardiac death (SCD) in comparison to the general population. Epidemiological data suggests that 0.1%-0.3% of the general population have electrocardiographic (ECG) findings suggesting that during sinus rhythm, in addition to atrioventricular (AV) conduction over the AV node-His bundle pathway, there is an additional atrioventricular conduction across an accessory pathway. Whilst in most cases, such phenomenon is associated with WPW syndrome, other similar conditions, including Lown-Ganong-Levine (LGL) syndrome and Mahaim-type pre-excitation, have also been documented. Our patient is a young man in his late twenties admitted with broad complex tachycardia at 252 beats per minute associated with diaphoresis and pre-syncope. In our case report, we describe how we managed this emergency, eventually unveiling the underlying aetiology as well as a stepwise approach to dealing with adult broad-complex tachycardia.

## Introduction

This is a case of a 29-year-old man admitted with palpitations associated with diaphoresis and pre-syncope. Broad-complex tachycardia at 252 beats per minute was documented with a right bundloid morphology. He was chemically cardioverted with intravenous (IV) amiodarone and an electrocardiogram (ECG) in sinus rhythm revealed pre-excitation with delta waves matching the direction of his QRS morphology in tachycardia. He was then referred for electrophysiology studies (EPS) that revealed an accessory pathway, which was subsequently successfully ablated. This report describes how a case of a new onset broad complex tachycardia of unclear aetiology was clinically managed consequently leading to a full diagnosis and successful treatment of Wolff-Parkinson-White (WPW) syndrome. Patients with WPW syndrome may experience chest pain, palpitations, dizziness, syncope and congestive heart failure. However, in much rarer instances, the first and only manifestation of the disease is sudden cardiac death (SCD) [1].

## Case presentation

We present a young patient under 30 years of age who attended our emergency department with symptoms of palpitations, pre-syncope and diaphoresis. He stated that over the last three years, he had been having infrequent palpitations, each lasting less than a minute, with no other associated symptoms. However, on this occasion, he reported feeling slightly light-headed but denied chest pain and breathlessness and was not noted to have collapsed or lost consciousness. Upon review of his initial ECG, he was immediately admitted to the resuscitation bay of the emergency department and promptly connected to a defibrillator machine. However, he showed no adverse haemodynamic signs or symptoms and remained stable on continuous cardiac monitoring.

A quick physical examination was carried out, which was somewhat reassuring, as the patient was fully conscious and alert, had no chest pain and no peripheral signs of heart failure. His heart sounds were clearly audible, pulses were present and jugular venous pulse (JVP) was not raised. He maintained a stable blood pressure in the range of 112/78mmHg and 118/80 mmHg, which was normal for him and oxygen saturation levels between 98% and 100%. A 12-lead electrocardiogram (ECG) was carried out to further assess the rhythm while he was commenced on IV Amiodarone.

**Figure 1:**
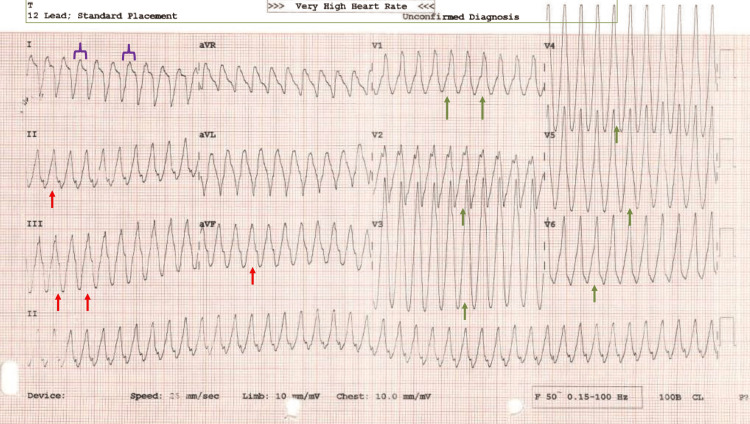
ECG at presentation that revealed a broad complex tachycardia at 252 beats/min, with a right bundloid morphology, positive in V1-V6 (green arrows), negative lead I (purple arrow bracket), and positive in II, III, and aVF (red arrows)

His electrocardiogram revealed a broad complex tachycardia at 252 beats/min, with a right bundloid morphology, positive in V1-V6, negative lead I, and positive in II, III, and aVF (Figure 1).

**Figure 2:**
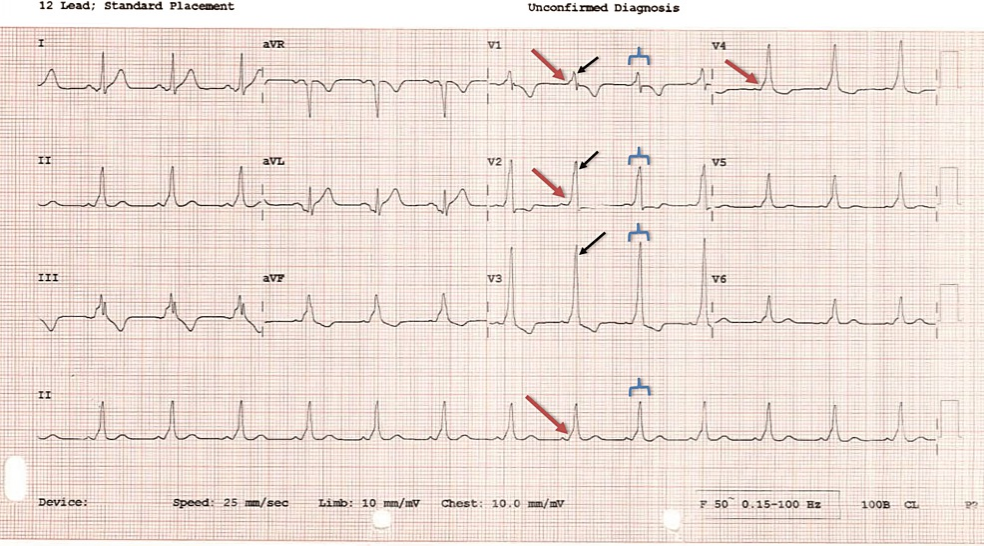
Electrocardiogram in sinus rhythm showing pre-excitation with delta waves (red arrows) that matched the direction of his QRS morphology (blue arrow bracket) in tachycardia. This is a left-sided antidromic (retrograde) accessory pathway due to the dominant R wave in V1-V3 (black arrows) and the presence of delta waves (red arrows), respectively.

**Figure 3:**
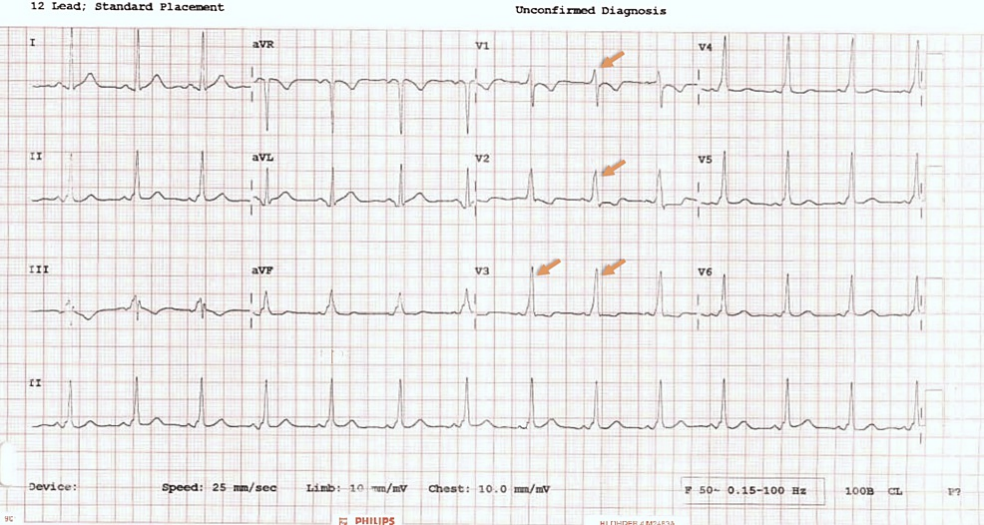
Electrocardiogram in sinus rhythm showing similar features as in Figure 2 Note the positive R wave in V1 (orange arrows), a pattern consistent with type A WPW syndrome (left accessory pathway). WPW: Wolff-Parkinson-White

Following the initial stat dose of IV Amiodarone infusion, he successfully cardioverted within minutes to sinus rhythm. His electrocardiogram in sinus rhythm showed pre-excitation with delta waves that matched the direction of his QRS morphology in tachycardia (Figures 2, 3). 

An impression was made of antidromic atrioventricular re-entrant tachycardia secondary to a left anterolateral accessory pathway. Subsequent ECG tracings remained suggestive of WPW with an aberrant pathway. He was admitted to the acute coronary unit for continuous telemetry and started on low-dose Bisoprolol (a relatively safe alternative) the following day to maintain a slower heart rate. He then had an inpatient referral for electrophysiology studies with or without ablation.

Upon further questioning, he reported having a family history consistent with premature cardiac diseases. His father had a myocardial infarction (MI) at the age of 54 and his mother’s cousin had sudden cardiac death (SCD) at 40 years (no clear diagnosis was made pre- or post-mortem). He denied the use of recreational stimulant drugs like cocaine and methamphetamine and did not consume alcohol excessively. The patient did not smoke and had never experienced any pre-syncopal or syncopal attacks in the past. He also mentioned being generally physically active and had never experienced exercise-induced cardiovascular events such as chest pain, palpitations or syncope in the past.

We then carried out further instrumental and blood investigations to look for possible abnormalities and triggers. His blood test results were as follows: haemoglobin level (Hb): 141g/L (130-180g/L), white cell count (WCC): 6.5 (normal: 4-11 x10*9/L), C-reactive protein of 2 mg/dL (normal: <5 mg/dL) - all of which were within normal limits. More importantly, his electrolytes results were completely normal (magnesium: 0.85 mg/dL (0.7 - 1 mg/dL), phosphate: 1.1 mmol/L (0.8 -1.50 mmol/L)), troponin I: 4.5 ng/L (< 14 ng/L), adjusted calcium: 2.56 mmol/L (2.12 - 2.63 mmol/L), thyroid-stimulating hormone: 1.38 mU/L (0.4 - 4.5 mU/L) - reference range included in brackets. All other blood test results were unremarkable.

Inpatient trans-thoracic echocardiogram (TTECHO) revealed borderline low left ventricular (LV) systolic function (ejection fraction between 50% and 55%), normal right ventricular (RV) function, mild mitral regurgitation, and tricuspid regurgitation.

Although we were able to find a likely underlying trigger based on his electrocardiogram in sinus rhythm, we still considered other possible differential diagnoses. The primary diagnosis at presentation in this case was regular broad complex tachycardia, which was immediately managed according to the ALS adult tachycardia algorithm [2]. From analysing the 12-lead ECG, we weren’t immediately able to identify that it was a pre-excited tachyarrhythmia at first glance. However, we had to look for the presence and/or absence of certain features to come to a reasonable conclusion. We agreed that based on the patient’s electrocardiogram, the key differentials to consider were ventricular tachycardia (mono- or polymorphic), supraventricular tachycardia with bundle branch block, atrial fibrillation with bundle branch block and pre-excited atrial fibrillation or flutter. 

Points that could occasionally point towards ventricular tachycardia include a regular broad-complex tachyarrhythmia, the presence of fusion and/or capture beats, with accompanying symptoms of cerebral hypoperfusion - none of which were present in our patient. Torsades, a type of polymorphic ventricular tachycardia, may be relatively well-tolerated by patients, and its ECG findings are mostly easier to interpret by acute physicians. This was not the case with our patient. In the case of atrial fibrillation with aberrancy (e.g. with bundle branch block or pre-excitation) as well as other pre-excited wide-complex tachyarrhythmias, previous electrocardiograms and patient history are usually crucial to making an accurate diagnosis [3].

In terms of the aetiology, there are many possible underlying mechanisms to consider depending on the type of arrhythmia. These may include congenital, structural, ion channel/electrical heart disorders or ischaemic heart diseases (less likely in this case given the age and risk profile of the patient). Cardioverting our patient with IV amiodarone was very useful in revealing the underlying features of Wolf-Parkinson-White on a much slower electrocardiogram in sinus rhythm [3].

Nevertheless, in the absence of adverse clinical features and previous electrocardiograms and due to the acute nature of the patient’s presentation, IV amiodarone was deemed the safest option.

In terms of treatment strategy and in the acute setting such as in this case, the patient was managed as a case of stable wide-complex (broad complex) tachycardia with no life-threatening features. According to the latest United Kingdom Resuscitation Council guidelines, life-threatening features include symptoms suggestive of ongoing myocardial ischaemia, shock, syncope, and severe heart failure [4]. In the absence of the above features, the patient had IV amiodarone for chemical cardioversion, which was successful. We then admitted the patient under cardiology and referred him for further electrophysiological study (EPS) and ablation with the findings outlined below.

The procedure was performed under local anaesthesia with sedation. Four ultrasound-guided punctures were made of the right femoral vein. A decapolar catheter was advanced to the coronary sinus, and quadripolar catheters to the right ventricular apex, His region, and high right atrium. 

Retrograde conduction was concentric and decremental. Antegrade conduction was decremental with manifest pre-excitation. Non-sustained tachycardia could be induced in the baseline state with single atrial extra stimuli with a morphology that was identical to his maximally pre-excited QRS during antegrade pacing and his clinical tachycardia. The tachycardia was non-sustained so no further diagnostic manoeuvres could be performed, but all evidence supported the clinical suspicion of antidromic (retrograde) AVRT.

CARTO® - a non-fluoroscopic 3D electro-anatomic mapping technique - was used to create a geometry of the left atrium using a D/F smart-touch catheter. The ventricular insertion of the accessory pathway was mapped. Ablation at the site of earliest ventricular activation abolished pre-excitation. A total of 2 minutes of 40W radio frequency was delivered around this area, targeting an ablation index of 550. Pathway conduction remained blocked during a 20-minute waiting period. The case was terminated at this point. The sheaths were pulled, and a Z-suture was deployed. There were no immediate complications.

**Figure 4:**
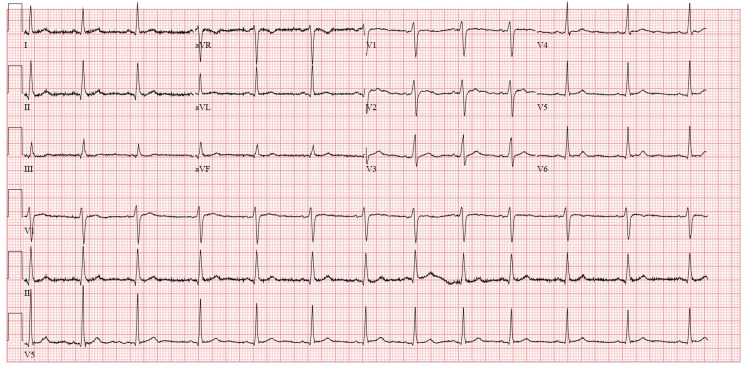
Electrocardiogram (ECG) Day 0 post-ablation showing the resolution of Delta waves

Radiofrequency ablation (RFA) was successful in this case as partly evidenced by the ECG taken just post-ablation on the day he had the procedure done (Figure 4).

This together with the outcome of the electrophysiology study suggests that the WPW pathway had indeed been successfully ablated. It therefore further strengthens the available evidence of up to 95% success and cure rate from radiofrequency ablation of accessory pathways [4].

Our patient was discharged the same day and advised to refrain from driving for two days and from heavy lifting or sports for one week. He was sent home on aspirin 300 mg daily for four weeks for thromboprophylaxis and asked to stop taking Bisoprolol. Upon initial follow-up four weeks post-ablative therapy, our patient had managed to fully resume work and other activities of daily living not being limited by any palpitations. He reported being symptom-free the entire period and now plans to return to the gym although aware not to strain excessively. 

**Figure 5:**
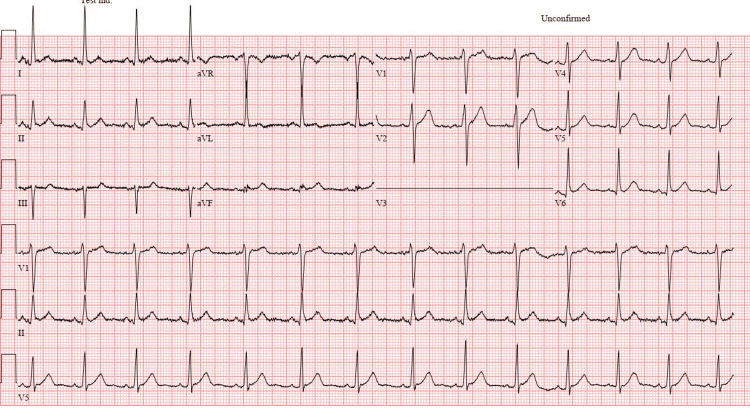
Electrocardiogram (ECG) four months post ablation showing the total resolution of Delta waves

Four months later, he was seen in the clinic and a repeat ECG was done, which showed full resolution of the accessory pathway (Delta wave) (Figure 5). He also recounted staying completely asymptomatic since the ablation was done and is now back to his usual daily routine without any limitations.

## Discussion

In this case, it became apparent a few days into his admission that this gentleman had initially attended a cardiology clinic six years ago in his early twenties following a general practitioner (GP) referral. At that time, he was referred to the cardiology service due to a four-month history of intermittent chest pains and an incidental finding of the Wolff-Parkinson-White pathway. This was followed by further extensive investigation with an exercise tolerance test (ETT), computed tomography-coronary angiography (CTCA), cardiac magnetic resonance (CMR) imaging with coronary artery tagging, and trans-thoracic echocardiography (TTEE), all of which concluded that he only had the pathway and not the syndrome. He was therefore discharged from the follow-up clinic and asked to only re-present if symptoms developed in the future. Due to the acuteness of his presentation as well as being very anxious, the patient could not recall these details at the time of admission.

The WPW syndrome affects 0.1-0.3% of the general population[5,6]. The main pathophysiology of this condition is manifested in the presence of an accessory pathway called the bundle of Kent. The bundle of Kent is a remnant of the embryological development of the heart that can conduct electrical impulses from atria to ventricles (anterograde conduction) and/or vice versa (retrograde conduction). The accessory pathway does not possess the unique ability to delay or slow down impulses coming from the atria or ventricle as does the atrioventricular (AV) node. This could pose a serious danger during supraventricular tachyarrhythmias, as the bundle of Kent may turn into a fast conduit. The delta wave seen on an ECG represents anterograde (orthodromic) conduction via the bundle of Kent and might therefore be easily missed in patients that only have intermittent conduction via this pathway or in those that conduct retrogradely (i.e bottom to top) [5]. According to studies, between 60% and 75% of accessory pathways are able to conduct in both directions (antegradely and retrogradely) between the atrium and ventricle. However, less than 35% only possess the ability to conduct in one direction, i.e., retrograde (from ventricle to atrium) [7].

WPW patients are generally at higher risk of developing atrial fibrillation later in life, reaching a probability of around 30%. One of the main mechanisms of sudden cardiac death in patients with WPW is believed to be through the conduction of rapid irregular atrial impulses via the described pathway that degenerates into life-threatening and potentially fatal ventricular tachyarrhythmias [8,9]. This patient, despite being cleared a few years ago, most certainly now has the syndrome, which before radiofrequency ablative treatment, was at higher risk of adverse events when compared to asymptomatic subjects. 

Risk stratification is done using a combination of both noninvasive and invasive electrophysiology (EPS) testing. This is done by evaluating the anterograde atrioventricular conduction rate over the accessory pathway looking for certain risk features. High-risk features on clinical evaluation include male sex, WPW pattern detected in the first two decades of life, history of atrial fibrillation (AF), arrhythmic symptoms (especially syncope), congenital heart disease (e.g., Ebstein's anomaly), or familial WPW syndrome. An essential point to consider is the patient’s occupation and day-to-day activities. Bus drivers or people who participate in competitive sports for instance are considered high-risk [5,6,10]. Of the life-threatening events, it has been reported that about 40% of cases occur at rest and about 45% of cases occur during activity [11]. The measurement of the shortest pre-excited R-R interval (SPERRI) is used to determine accessory pathway properties in an invasive electrophysiology study. A SPERRI of 220 to 250 ms, especially less than 220 ms, is more commonly seen in patients with WPW who have experienced cardiac arrest. Thus, SPERRI less than 250 ms may be considered as an indication for consideration of ablation. All of these options should involve a careful discussion of the risks/benefits with the patient and their family [12,13].

Furthermore, it is important to note that certain medications that slow down the AV node (e.g., Adenosine, Verapamil, Digoxin, and Diltiazem) are generally contraindicated in an acute setting in patients with broad complex tachycardia (BCT) suspected to have an accessory pathway. This patient was chemically cardioverted to sinus rhythm with the use of IV Amiodarone as one of the safer options due to reasons already mentioned and in line with Resuscitation Council UK and European Society of Cardiology (ESC) guidelines for stable broad complex tachyarrhythmias [2]. Other options to consider in chemical cardioversion of wide complex tachycardia are Lidocaine and Procainamide. Lidocaine should notably be avoided here due to strong evidence of underlying pre-excitation syndrome and is broadly avoided in patients with WPW.

## Conclusions

This case provides various insights into the management of WPW disease. Key lessons to take away are discussed below. First, when faced with broad (or wide) complex tachycardia, it is imperative to follow the recommended guidelines regarding the management of wide complex tachycardia. Direct current (DC) cardioversion is recommended (Class IA) if unstable, whereas in stable cases, the first choice should be Amiodarone as most other rate-limiting medications could precipitate fatal arrhythmias; especially if one is unsure or has no access to baseline ECG to assess whether a patient has an accessory pathway.

Furthermore, accessing a baseline ECG may be crucially worthwhile as demonstrated in our case study, where an electrocardiogram in sinus rhythm confirmed our suspicion and informed our decision on how to proceed further. However, in this case, going by the adult advanced life support (ALS) algorithm for managing acute wide complex tachycardia proved essential to identifying the underlying rhythm. Whilst a WPW pathway could remain quiescent throughout life, it also has the potential to progress to WPW syndrome as was seen in our patient. A long-term solution to this is RFA, which when done by an experienced specialist can cure WPW syndrome with excellent success rates.
